# Analysis of Dyssynchrony and Ventricular Function in Right Univentricular Stimulation at Different Positions

**DOI:** 10.21470/1678-9741-2017-0056

**Published:** 2017

**Authors:** Ana Paula Susin Osório, Stefan Warpechowski Neto, Antonio Lessa Gaudie Ley, Marcelo Haertel Miglioranza, Laura Lessa Gaudie Ley, Eduardo Dytz Almeida, Roberto Tofani Sant'anna, Tiago Luiz Luz Leiria

**Affiliations:** 1 Programa de Pós-graduação do Instituto de Cardiologia do Rio Grande do Sul/Fundação Universitária de Cardiologia, Porto Alegre, RS, Brazil.; 2 Pontifícia Universidade Católica do Rio Grande do Sul (PUCRS), Porto Alegre, RS, Brazil.

**Keywords:** Pacemaker, Artificial, Ventricular Dysfunction, Heart Failure, Stroke Volume

## Abstract

**Introduction:**

Chronic stimulation of the right ventricle with pacemaker is associated with ventricular dyssynchrony and loss of contractility, even in subjects without previous dysfunction. In these patients, there is a debate of which pacing site is less associated with loss of ventricular function.

**Objective:**

To compare pacemaker-induced dyssynchrony among different pacing sites in right ventricular stimulation.

**Methods:**

Cross-sectional study of outpatients with right ventricle stimulation higher than 80% and preserved left ventricular ejection fraction. Pacing lead position (apical, medial septum or free wall) was assessed through chest X-rays. Every patient underwent echocardiogram to evaluate for dyssynchrony according to CARE-HF criteria: aortic pre-ejection time, interventricular delay and septum/posterior wall delay on M mode.

**Results:**

Forty patients were included. Fifty-two percent had apical electrode position, 42% mid septum and 6% free wall. Mean QRS time 148.97±15.52 milliseconds. A weak correlation between the mean QRS width and pre-aortic ejection time (r=0.32; *P*=0.04) was found. No difference in QRS width among the positions could be noted. Intraventricular delay was lower in apical patients against mid septal (34.4±17.2 *vs.* 54.3±19.1 *P*<0.05) - no difference with those electrode on the free wall. No difference was noted in the pre-aortic ejection time (*P*=0.9).

**Conclusion:**

Apical pacing showed a lower interventricular conduction delay when compared to medial septum site. Our findings suggest that apical pacing dyssynchrony is not ubiquitous, as previously thought, and that it should remain an option for lead placement.

**Table:** 

Abbreviations, acronyms & symbols	
ACE	= Angiotensin enzyme converter
AF	= Atrial fibrillation
CAD	= Coronary artery disease
DM	= Diabetes mellitus
ECG	= Electrocardiography
HF	= Heart failure
LVEF	= Left ventricular ejection fraction
NYHA	= New York Heart Association
RV	= Right ventricle
RVFW	= Right ventricle free wall
RVMS	= Right ventricle medium septum
RVOT	= Right ventricle outflow tract

## INTRODUCTION

Apical pacing of the right ventricle (RV) has been the preferred site for pacemaker lead positioning^[1,2]^. However, data on long-term follow-up revealed potentially harmful effects of chronic RV apical stimulation^[3-5]^. The mechanisms involved in such effects include myofibrillar derangement and intraventricular and interventricular dyssynchrony, which may lead to loss of contractile function^[6]^. The clinical consequence of such changes manifest through worsening of left ventricular ejection fraction (LVEF) and, in some cases, heart failure (HF) symptoms. Once LVEF starts to fall, it leads to deterioration in quality of life, to an increase in atrial fibrillation burden and to a tendency to a higher mortality^[7-11]^. The DAVID trial^[12]^ showed that RV pacing is poorly tolerated by patients and may cause heart failure. The BLOCK-HF^[13]^ study showed that apical RV stimulation associates with poor outcomes in patients with atrioventricular block and mild to moderate HF.

Recent studies suggest that non-apical pacing sites may reduce dyssynchrony and consequently preserve LVEF^[14]^. However, these studies were limited by a relatively small sample size and heterogeneity of both the selected population and of the methods used for monitoring for LV function^[15]^. As such, the ideal pacing site for patients requiring RV-only pacing, if existent, remains a matter for debate^[16]^.

In this study, LVEF and ventricular synchrony criteria were compared (as adopted in the CARE-HF trial)^[17]^ in patients with different RV pacing sites.

## METHODS

This was a cross-sectional study conducted at the pacemaker clinic of our institution between June and November 2015. Patients aged 18 years or older were eligible if they had a RV pacing percentage above 80 and a LVEF above 50%. Exclusion criteria were: atrial fibrillation (AF), New York Heart Association (NYHA) HF symptoms class III our IV, moderate to severe valvular disease, epicardial electrode, biventricular pacemaker and HF hospitalization before pacemaker implant. All participants signed the consent form. Study protocol was approved by local ethics committee.

During the index visit, clinical data and current medication use were assessed. Clinical data collected were the reason for pacemaker implant, pacemaker program mode, RV pacing percentage, height, weight, resting heart rate (HR), NYHA HF functional class status and the presence of comorbidities such as hypertension, diabetes mellitus (DM), coronary artery disease (CAD), dyslipidemia and smoking status. Relevant medications assessed were diuretics, angiotensin enzyme converter (ACE) inhibitors, beta-blockers, calcium channel blockers and amiodarone.

All patients underwent 12-lead electrocardiography (ECG) examination, chest X-ray and transthoracic echocardiography. ECG finding collected was largest QRS duration of the 12 leads. X-ray was used to assess RV lead position^[18]^, which was divided into 3 categories: RV apex, middle RV septum and RV free wall.

Echocardiography was performed using Vivid E9 machine (GE Healthcare) and EchoPAC software (GE Healthcare). Single and two-dimensional images were obtained, as were Doppler velocities and pulsed and continuous Doppler tissue imaging^[19]^. All the analyses were performed after, at least, three months from the date of implantation. The following measurements of dyssynchrony and ventricular function were analyzed^[17]^:

- Driving delay between the interventricular septum and posterior wall in M mode (cutoff ≥ 130 ms);
- Difference between pre-ejection times the for the left and right ventricles (interventricular delay) and the pulsed Doppler (cutoff ≥ 40 ms);
- Measure the pre-ejection time LV (aortic) the pulsed Doppler (cutoff ≥ 140 ms);
- Measurements of systolic and diastolic left ventricular volume and left atrial volume in the biplane method;
- Left ventricular ejection fraction calculation using the Simpson biplane method;
- Ratio E/e ' by Doppler analysis of transmitral and pulsed Doppler flow.

### Statistical Analysis

The collected data were stored in Excel spreadsheets and analyzed using the softwares SPSS version 23.0 (IBM Corp. Released 2013. IBM SPSS Statistics for Windows, Version 22.0. Armonk, NY: IBM Corp.) and MedCalc version 8.2 (MedCalc Software bvba, Ostend, Belgium; https://www.medcalc.org; 2008). Continuous variables were expressed as mean and standard deviation or median and interquartile range. Categorical variables were presented as absolute and percentage number.

Bivariate comparisons were made with chi-square test or test-T-tailed, as appropriate.

For correlation analysis between the QRS length and CARE-HF echocardiographic indexes the Spearman coefficient (rs) was used. The data were transformed into rank to analyze the differences between the obtained values and the standard error. A *P*<0.05 was considered statistically significant.

## RESULTS

A total of 40 patients were included for this analysis. Other two patients able to participate were excluded because they did not have a post-implant chest X-ray recorded on our electronic chart. The majority (55%) of patients were male and their mean age was 69 years (Table 1). The most common pacemaker indication was complete AV block (62.5%). Hypertension was the most prevalent comorbidity and only 12.5% of patients had mild HF symptoms (NYHA functional class II). No patient had post procedure cerebrovascular event. Mean heart rate was 71 beats per minute and mean largest QRS on ECG was 148.97±15.52 ms. Mean RV pacing percentage was 94.95% and most pacemakers (72.5%) were on DDD mode.

**Table 1:** Clinical and pacing echocardiographic characteristics (n=40).

Variables	N (%) or mean ± SD
Age	69.25±14.76 years
Male	22 (55%)
BMI	27.91±4.78
Heart rate (bpm)	71.15±9.78
QRS in lead DII during echo (ms)	93.68±15.72
Largest QRS on 12 lead ECG	148.97±15.52
Hypertension	26 (65%)
DM	10 (25%)
Dyslipidemia	13 (32%)
Smoking	13 (32.5%)
CAD	6 (15%)
NYHA functional class	
I	87.5%
II	12.5%
Current medications	
Diuretics	15 (37.5%)
ACE inhibitors	12 (30%)
Angiotensin receptor blockers	11 (27.5%)
Beta-blockers	20 (50%)
Calcium channel blockers	5 (12.5%)
Amiodarone	1 (2.5%)
Reason for the Implant	
Complete AV block	25 (62.5%)
2^nd^ degree AV block	7 (17.5%)
Others	8 (20%)
Stimulation Mode	
DDD	29 (72.5%)
DDDR	6 (15%)
VVIR	4 (10%)
VDD	1 (2.5%)
RV pacing (%)	94.95%±10.65%

Bpm=beats per minute; ms=milliseconds; DM=diabetes mellitus; CAD=coronary artery disease; NYHA=New York Heart Association; ECG=electrocardiography; ACE=angiotensin enzyme converter, BMI= body mass index; AV=atrioventricular; RV=right ventricle

Regarding echocardiographic evaluation, a mean LVEF of 63% was found. QRS measurement during echocardiogram ranged from 77.96 to 109.4 ms, averaging 93.68 ms on lead DII. As for dyssynchrony parameters, the pre-aortic ejection time was slightly prolonged with a mean of 141 ms, and the interventricular delay measurement was also extended, averaging 43 ms (Table 2).

**Table 2:** Echocardiographic measurements of ventricular synchrony, in milliseconds.

Ventricular Synchrony Measurements	Mean ± SD
Aortic pre-ejection time	140.60±22.52
Pulmonary pre-ejection time	98.33±22.12
Interventricular delay	43.27±20.25
Septum/posterior wall delay (M mode)	105.50±46.9
Diastolic volume of LV (ml)	91.68±29.08
Systolic volume of LV (ml)	34±13.10
Ejection fraction (%)	63.50±6.09
Left atrial volume (ml)	32.12 ±10.24
Ratio E/e'	11.11±4.50

LV=left ventricle

### Pacing Site Comparisons

**Fig. 1:**
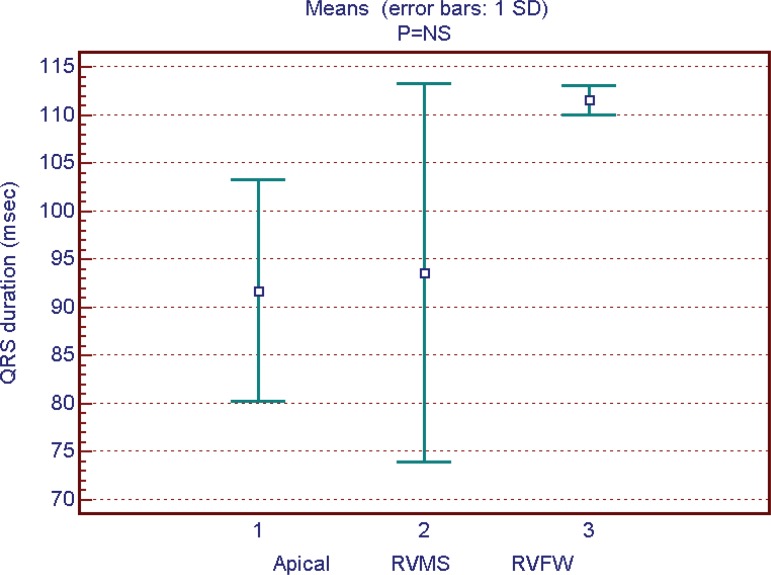
QRS on lead DII during echocardiogram (ms). RVFW=Right ventricle free wall; RVMS=Right ventricle medium septum

**Fig. 2:**
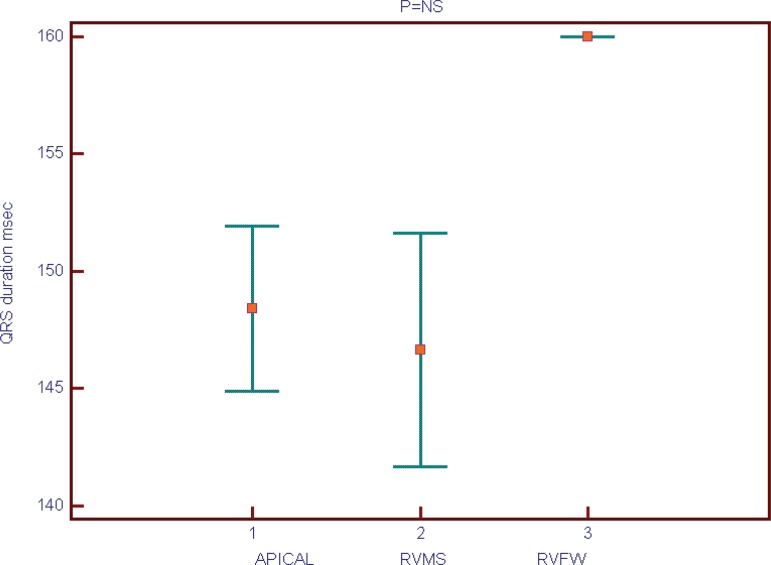
Largest QRS on 12 lead ECG (ms). RVFW=right ventricle free wall; RVMS=right ventricle medium septum

**Fig. 3:**
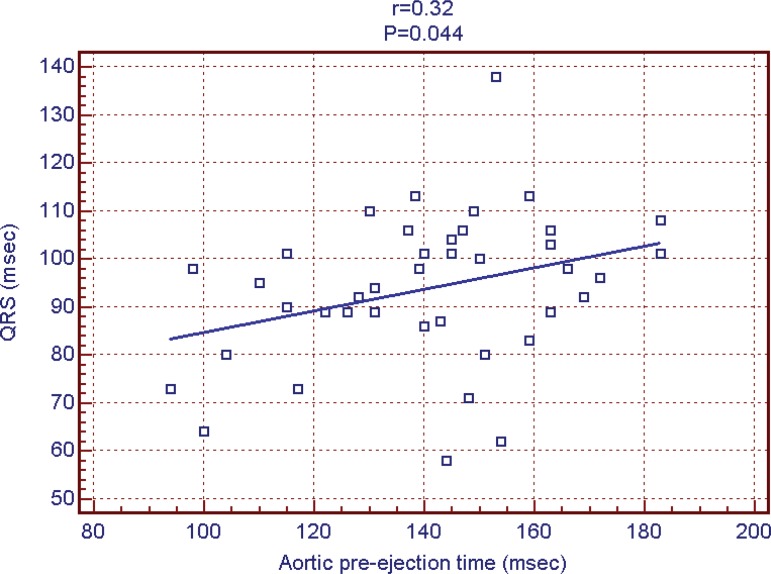
QRS duration and aortic pre-ejection time.

**Fig. 4:**
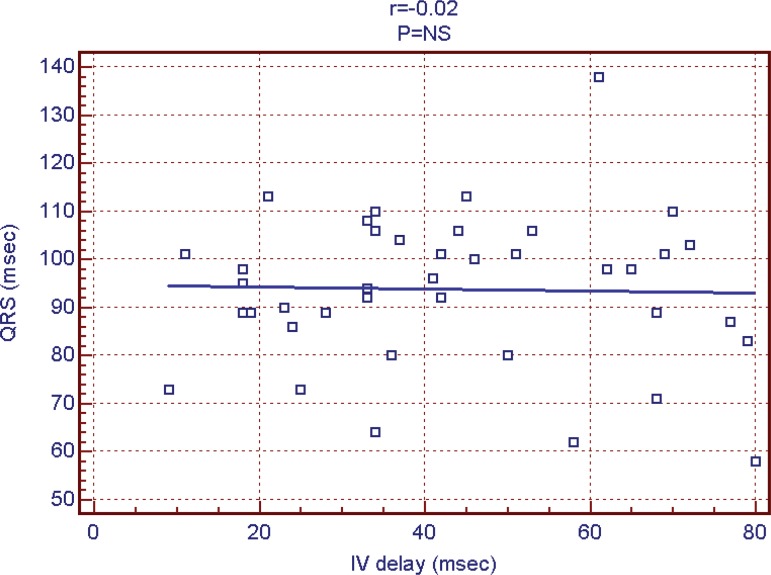
QRS duration and interventricular delay.

**Fig. 5:**
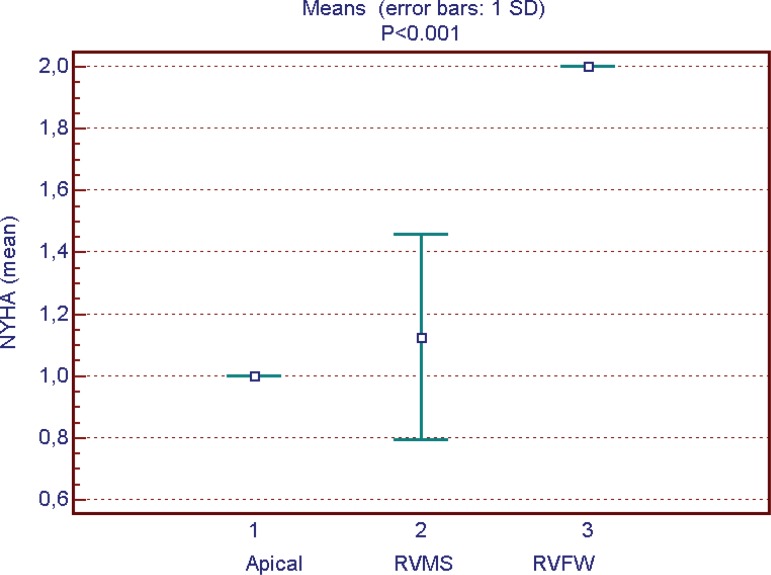
New York Heart Association class and lead position. NYHA=New York Heart Association; RVFW=right ventricle free wall; RVMS=right ventricle medium septum

**Fig. 6:**
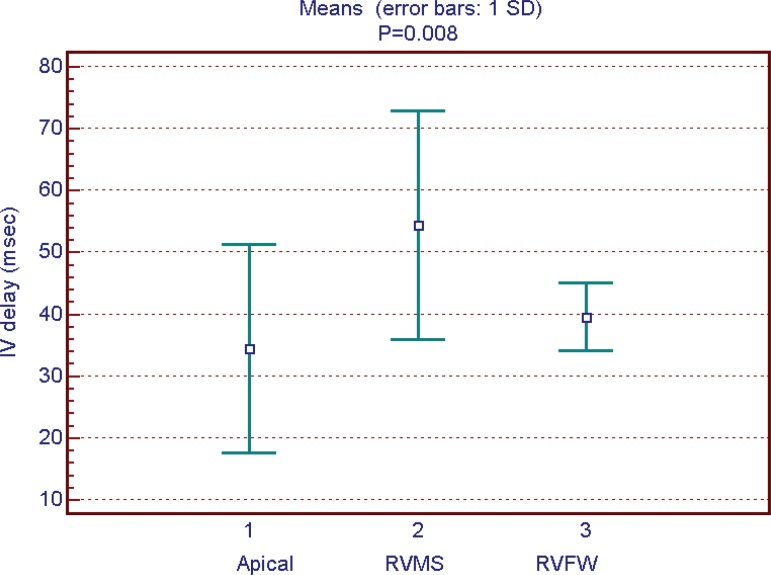
Intraventricular delay and lead position. IV=interventricular; RVFW=right ventricle free wall; RVMS=right ventricle medium septum

**Fig. 7:**
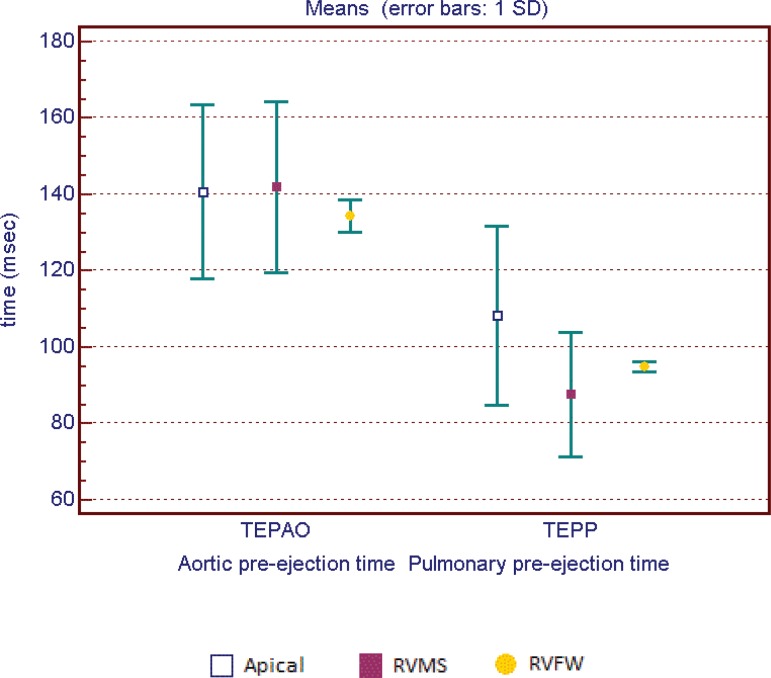
Aortic pre-ejection time and pulmonary pre-ejection time according to pacing site.

Apical RV lead position was found in 52% of patients, RV medium septum (RVMS) in 42% and RV free wall (RVFW) in the remaining 6%. No differences in QRS duration between the 3 groups could be noted (Figures 1 and 2). Mean QRS duration had a weak correlation with pre-aortic ejection time (r=0:32; *P*=0.044) (Figure 3) and had no correlation with interventricular delay (Figure 4). NYHA average was lower in patients with apical stimulation *(P*<0.001) (Figure 5). Intraventricular delay was lower in those with apical RV pacing when compared to those with RVMS pacing (34.4±17.2 ms *vs.* 54.3±19.1 ms; *P*<0.05), and no difference was found when compared to those with RVFW pacing (Figure 6). Pre-aortic ejection time did not differ among the groups (*P*=0.9) (Figure 7).

## DISCUSSION

**Fig. 8:**
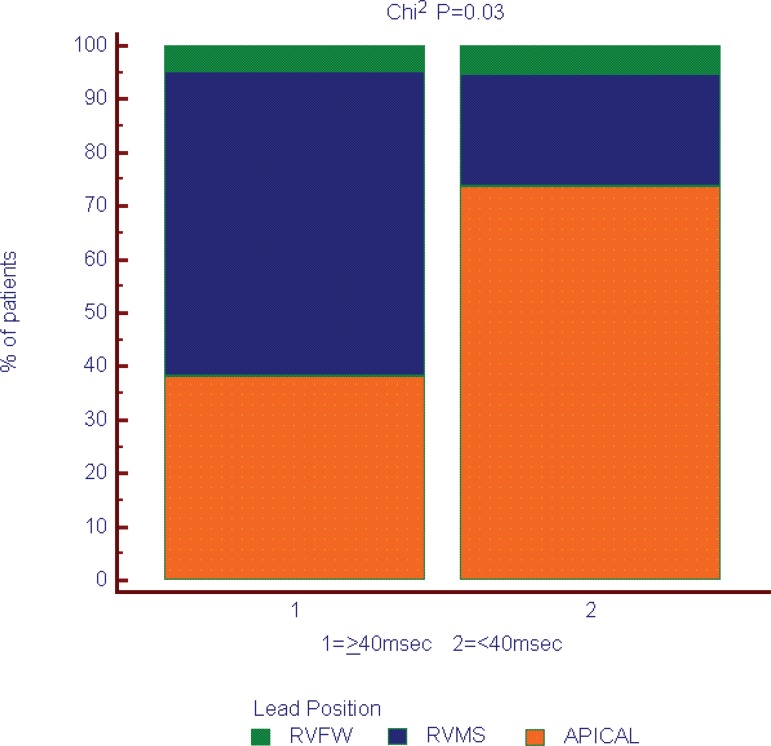
Interventricular dyssynchrony and pacing position.

In this study, left ventricular function and myocardial synchrony were compared among different right ventricular pacing sites. Our main finding was that RV apical pacing was related to a lower degree of interventricular dyssynchrony when compared to septal pacing (Figure 8). Furthermore, we found that QRS duration did not correlate with pacing site and correlated poorly with dyssynchrony parameters.

Our findings are in contrast with several previous studies that have suggested that RVMS might provide better results when compared to RVA pacing^[20-23]^. It has been shown that apical pacing potentially leads to electrical dyssynchrony, and that it changes the physiological apex-to-base contraction to an alternate pattern^[24]^. Theoretically, RVMS pacing could create a faster depolarization wavefront, since it is in close proximity with the normal conduction system. However, there are still no conclusive data to prove that these changes translate into clinical outcomes^[25]^.

One of such studies showed that septal pacing not only did not correlate with LVEF worsening, but also had fewer atrial arrhythmias during the follow-up period^[11]^. It should be noted, however, that those patients had a lower percentage of RV pacing when compared to subjects in our study (50-60% *vs.* > 80%). A second study has shown that septal pacing relates to improvement in 6-minute walk test^[23]^, even though the apical position presented the same result. On the other hand, another study in elderly patients has shown that even though apical pacing was related with worsening of LVEF, this finding did not translate into heart failure symptoms^[2]^. Recent review suggests that unfavorable clinical outcomes are associated not only with the stimulation site, but also with other pre-existing condition as CAD and systolic dysfunction where the compensatory mechanisms of dyssynchrony has been exhausted^[5]^.

The heterogeneity both of dyssynchrony criteria and of patient selection across these studies interferes with extrapolations and hampers pooled-data analysis. In fact, a metaanalysis on this subject has highlighted the diversity of study populations and inclusion criteria concluding that non-apical pacing sites were non-inferior to apical ones, with a statistical tendency to be better^[14]^. In this review, 14 randomized clinical trial analyzed population ranging from 12 to 122 people, followup periods of 3 to 90 months, different evaluation methods (echocardiography and nuclear imaging) and different cutoff points for ventricular function as selection criteria; some of them had LVEF as low as 27%. Inclusion of patients with AF and very broad definitions among the control and study groups by percentage of stimulation below and above 10%, respectively, contribute even more to the heterogeneity of the results, whose clinical translation is still uncertain.

Not evaluating RV outflow tract (RVOT) pacing is a potential limitation of our study. Even though there are several studies that have suggested this pacing site as a good alternative to RVA, our institution does not place leads in that position routinely. Intraventricular flow was not evaluated either, which has been shown to markedly change in RVA pacing. However, since the clinical meaning of such measurement is yet unknown, CARE-HF dyssynchrony criteria was chosen to be used in our study, hopefully to allow study comparisons and pooled-data analysis.

## CONCLUSION

In this study, RVA pacing showed a lower interventricular conduction delay when compared to RVMS pacing. Our findings suggest that RVA pacing dyssynchrony is not ubiquitous as previously thought, and that it should remain an option for lead placement.

**Table:** 

Authors' roles & responsibilities	
APSO	Actively participated of literature review, article review, interpretation of results and approved the final version; performed the echocardiographic evaluation; final approval of the version to be published
SWN	Actively participated of literature review, article review, interpretation of results and approved the final version; final approval of the version to be published
ALGL	Actively participated of literature review, article review, interpretation of results and approved the final version; final approval of the version to be published
MHM	Actively participated of literature review, article review, interpretation of results and approved the final version; final approval of the version to be published
LLGL	Actively participated of literature review, article review, interpretation of results and approved the final version; final approval of the version to be published
EDA	Actively participated of literature review, article review, interpretation of results and approved the final version; final approval of the version to be published
RTS	Acquisition, analysis, or interpretation of data for the work; final approval of the version to be published
TLLL	Acquisition, analysis, or interpretation of data for the work; final approval of the version to be published
